# Successful Anesthetic Management of a Pediatric Patient With Wolf-Hirschhorn Syndrome Undergoing Strabismus Surgery: A Case Report

**DOI:** 10.7759/cureus.70099

**Published:** 2024-09-24

**Authors:** Linda Gholmieh, Rosanne El Assaad

**Affiliations:** 1 Anesthesiology, Lebanese American University Medical Center, Beirut, LBN

**Keywords:** airway management, pediatrics, strabismus, sugraglottic device, wolf-hirschhorn

## Abstract

Wolf-Hirschhorn syndrome (WHS) is a rare genetic disorder caused by 4p chromosome microdeletion, characterized by distinctive craniofacial features, growth delay, short stature and slow height gain, variable degrees of intellectual disabilities, epilepsy, and congenital heart disease. Anesthetic management in pediatric patients with WHS requires a tailored approach due to the complex multisystem involvement, and the optimal strategy remains undetermined despite several published case reports. We present the airway management of an eight-year-old patient with WHS undergoing strabismus surgery using a supraglottic device. Although our patient’s distinctive craniofacial features of WHS suggested a potential for difficult intubation; after mitigating the risk of aspiration, a flexible laryngeal mask was successfully placed on the first attempt, offering a viable alternative to the challenges of tracheal intubation.

## Introduction

Wolf-Hirschhorn syndrome (WHS) is a rare congenital disorder caused by microdeletion of the short arm of chromosome 4 (del 4p16.3) [1]. These patients have distinctive phenotypic features, the most striking is their craniofacial features, represented by the wide bridge of the nose continuing to the forehead (the so-called “Greek warrior helmet appearance”) [2]. Other associated phenotypic and clinical characteristics include intrauterine growth retardation and later on short stature, low weight, hypotonia, intellectual disability, epilepsy, skeletal anomalies, congenital heart defects (septal defects and pulmonary stenosis), eye abnormalities, hearing loss, genitourinary tract defects, and immunological disorders [3]. WHS is associated with a poor prognosis, with approximately 30% mortality in the first two years of life, either of cardiac failure or bronchopneumonia [4].

Patients with WHS may require multiple corrective surgeries under general anesthesia. However, the abnormalities that accompany the syndrome may present an anesthetic challenge. We hereby present the anesthetic management of an eight-year-old patient with WHS presenting for surgery.

## Case presentation

This is a case of an eight-year-old male patient with WHS, presenting for strabismus surgery. The patient was born full-term through normal vaginal delivery (NVD). His past medical history is significant for neonatal intensive care unit (NICU) admission for two weeks, requiring intubation due to his cleft palate, and a small atrial septal defect (ASD). His past surgical history is significant for cleft palate repair at four months of age, bilateral hernia repair, and bilateral tympanostomy tube placement, both at the age of seven years. All procedures were performed under general anesthesia without any complications. Since all previous surgical interventions were performed at another hospital, we were not able to review the patient's anesthesia charts as we did not have access to their medical records. Regarding his allergies, the patient is known to develop erythema to adhesive tape.

**Figure 1:**
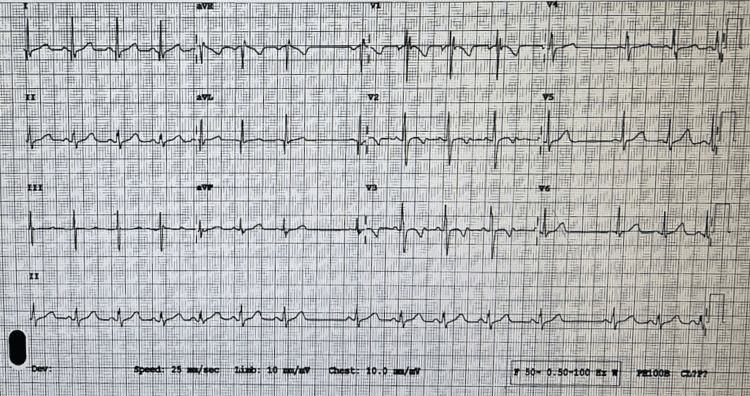
Electrocardiogram (ECG) showing sinus arrhythmia and borderline QTc prolongation

The preoperative assessment showed an active and cooperative child with syndromic features and micrognathia. Regular Laboratory tests were within normal range except for mildly low hemoglobin count and high red blood cell distribution width (RDW). The cardiac evaluation was negative for any cardiac murmur. An electrocardiogram (ECG) showed a sinus arrhythmia with borderline QTc prolongation (Figure 1). The patient's sinus arrhythmia was deemed nonpathological and did not require further evaluation.

A transthoracic echocardiography (TTE) showed a small patent foramen ovale (PFO). Accordingly, the pediatric cardiologist recommended using an in-line filter for intravenous (IV) line and avoiding medications that may cause QT prolongation.

The case was scheduled to be done as the first surgery, taking all malignant hyperthermia precautions, along with difficult set intubation made readily available in the operating room. The patient did not receive any premedication. In the operating room, standard American Society of Anesthesiologists (ASA) monitoring was applied, including non-invasive blood pressure, pulse oximeter, and 5 lead ECG. A 24-gauge intravenous (IV) catheter was inserted along with the in-line filter. The patient underwent intravenous anesthesia induction, 18mg lidocaine, 100mg propofol, and 20mcg fentanyl. When he became unresponsive, a number 2 laryngeal mask airway (LMA) Flexible™ (Teleflex Inc., Wayne, PA, USA) was inserted successfully on the first attempt without any complications. A rectal temperature probe was then placed and 3mg dexamethasone was administered intravenously. Anesthesia was maintained using total IV anesthesia (TIVA), propofol infusion at 60mcg/kg/min, and Remifentanil infusion titrated between 0.03 and 0.1 mcg/kg/min, titrated as needed based on heart rate and blood pressure variations. At the incision, there was a drop in the patient’s heart rate to 55 bpm, which was responsive to 0.2mg atropine. His vital signs remained stable throughout the entire procedure afterward. The procedure took 50 minutes. A 270mg paracetamol was given 10 minutes before the end of the procedure. Propofol and remifentanil infusions were terminated when the last suture was applied. The patient emerged smoothly after 10 minutes, and he was transferred to the post-anesthesia care unit (PACU) while still completely conscious. The patient was admitted to the hospital for 24-hour monitoring and was discharged after an uneventful stay.

## Discussion

Due to the craniofacial anomalies and short stature associated with WHS, airway management and endotracheal intubation may be difficult. Although some reported the use of a smaller tube size [4,5], yet direct laryngoscopy, with proper patient positioning, was enough to secure tube placement [6]. In our patient, we decided to insert an LMA to avoid the potential for difficult airway challenges in the absence of any risk for aspiration. Sari et al. published an unsuccessful insertion of an Igel after two attempts in an adult patient with WHS and severe kyphoscoliosis [7]. Due to the risk of failure, an endotracheal tube was opened and ready for use along with the kit for difficult intubation.

Congenital heart disease is considered common in patients with WHS. Our patient had a mild ASD, that was managed by careful titration of our anesthetic medications to maintain hemodynamic stability and placement of an in-line filter.

Although cases of malignant hyperthermia have been reported in WHS patients, the association between both has not been proven. Ginsburg and Purcell-Jones were the first to report a case of malignant hyperthermia in a 21-month-old girl with WHS who received succinylcholine and inhaled halothane intraoperatively [4]. Chen et al. were the first to report a case of delayed malignant hyperthermia in an eight-month-old female with WHS two hours after receiving halothane, succinylcholine, and atracurium [8]. Both cases were managed successfully with dantrolene and supportive care. Keeping that in mind, all malignant hyperthermia precautions were taken while managing our patient, the anesthesia machine was flushed the night before the operation, the procedure was performed as the first case to avoid any possible contamination from previous cases, all medications known to trigger malignant hyperthermia were removed from the operating room, weight appropriate dose of dantrolene was kept in the operating room, and patient's temperature was monitored continuously. TIVA was used. Although a possible delayed case of malignant hyperthermia was reported using TIVA by Choi et al., it was never proven [5]. Our patient was monitored up to 24 hours post-procedure and was discharged with an uneventful overnight stay.

## Conclusions

To our knowledge, this is the first case report describing the successful placement of a supraglottic device in a patient with WHS, while maintaining hemodynamic stability and avoiding the need for neuromuscular blocking agents. However, anesthesia should be individually tailored, as WHS patients can present with various abnormalities, each of which can influence their response to anesthesia differently.
